# Multiferroic ABO_3_ Transition Metal Oxides: a Rare Interaction of Ferroelectricity and Magnetism

**DOI:** 10.1186/s11671-019-2961-7

**Published:** 2019-04-24

**Authors:** Irfan Hussain Lone, Jeenat Aslam, Nagi R. E. Radwan, Ali Habib Bashal, Amin F. A. Ajlouni, Arifa Akhter

**Affiliations:** 1 Department of Chemistry, Faculty of Science Yanbu-30799, Taibah University, Al-Madina, Saudi Arabia; 2 Department of Chemistry, Taibah University, Al-Madina, 30002 Saudi Arabia; 3 Department of Botany, Faculty of Science, Punjabi University, Patiala, Punjab 147002 India

**Keywords:** Ternary metal oxides, Ferromagnetic, Ferroelectric, Multiferroic compounds, Bismuth ferrites, RMnO_3_, Rare earth metal oxides

## Abstract

This review article summarizes the development of different kinds of materials that evolved interest in all field of science particularly on new nano-materials which possess both electric and magnetic properties at the nanoscale. Materials of such kind possessing both magnetic and electric properties have tremendous applications and own an intensive research activity. These materials induce new properties which are particularly important in electronic and magnetic devices and even in the materials where magnetic property will change by electric field or vice versa. The discovery of such ferroic properties for scientific applications is the need of hour and spreads an exciting new area that has technical and commercial potential for the discovery of advanced materials. In recent studies, the actual path by which the multiferroic properties exist has been focused and new metal oxide compounds were discovered. The understanding of the structure of these compounds through research describes a wide range of applications and the challenges of these multiferroic materials that need to be explored. In this study, fundamental aspects and structural variations of ternary transition metal oxides have been covered which possess novel properties in storage devices such as hard disk platters and magnetic read heads.

## Introduction

Magnetic properties of objects at nanoscale range have been given the name of concept nanomagnetism with a prone area of research in all scientific fields. The properties and applications of magnetic nanoparticles, nanofilms, nanorods, and many more have been used earlier also in geology as ferrofluids and have enough scope to explore in the future [1]. These advanced materials have been used in other aspects, such as in loudspeakers and in the medical field for drug delivery [2] or even in magnetic hyperthermia [3]. The storage materials at very small size have usually found good efficiency if fabricated in small devices that reduces the dimension of machines. These small devices made up of magnetic nanoparticles play an important role in industries and most importantly in biomedical applications [4]. These materials have been applied to magnetic resonance imaging (MRI) devices that enable and visualize the local environment of tissue cells of cancer cells or tumors [5]. These magnetic nanoparticles have unique biomedical applications particularly to treat central nervous diseases and need to explore further to find innovative approaches in drug delivery to treat Central Nervous System (CNS) diseases [6].

**Fig. 1:**
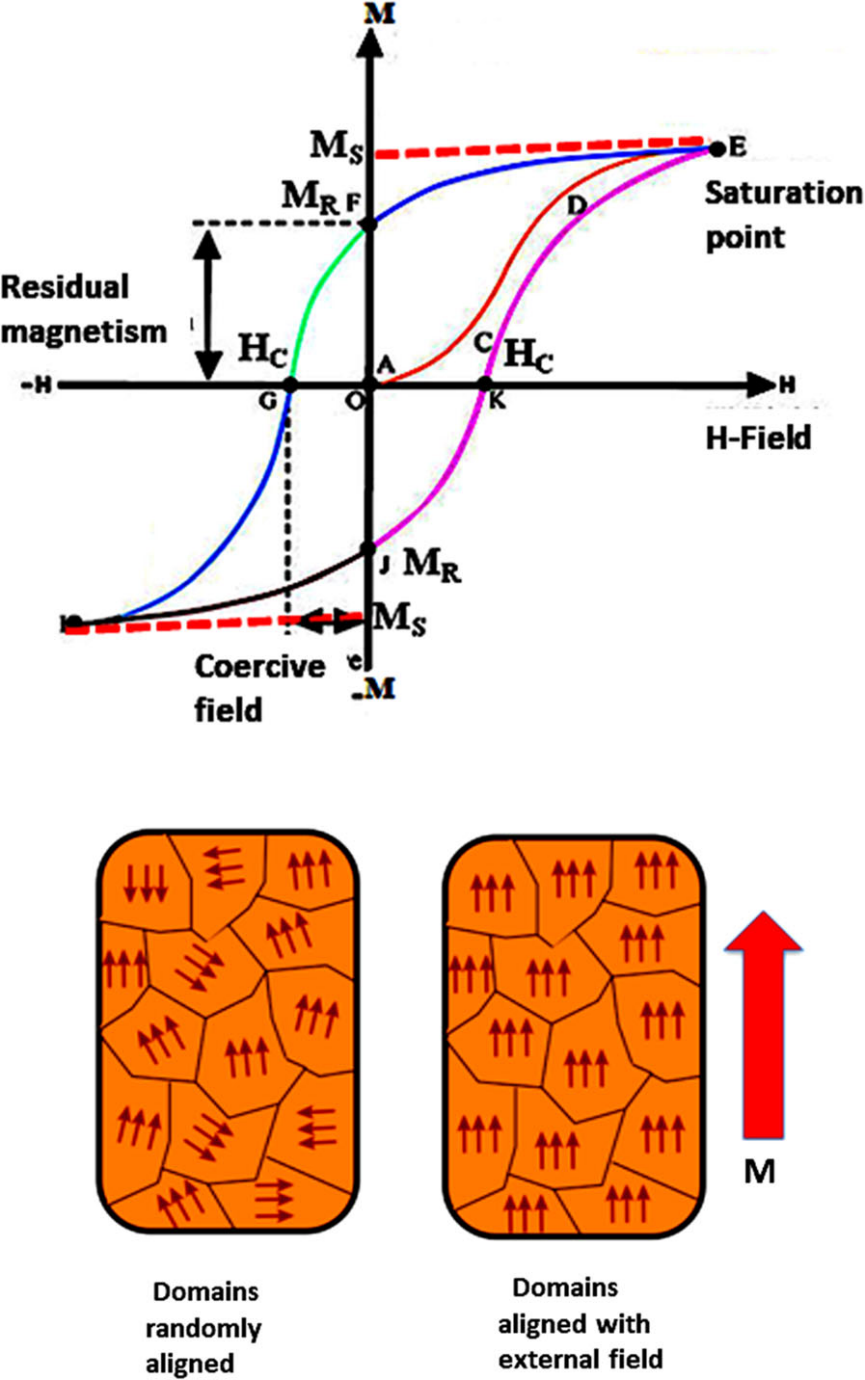
Ferromagnetic hysteresis loop and effect of magnetic domain alignments on applying magnetic field

Spontaneous magnetization can be created in a loop-like structure called hysteresis by the applied magnetic field. This particular feature of materials has given the name of ferromagnetic materials, and this property of materials originates from the electron spins and their orbital motion around the nucleus. In the absence of an external magnetic field, the magnetic moments are randomly oriented but when a field is applied, these spins are locked into a particular order and small group of spins to form domain-like structures. The structures and the typical hysteresis loop of these magnetic materials are shown in Fig. 1. Transition metals like nickel, cobalt, chromium, and iron have magnetic moments originating from spin orientations and also have an orbital contribution to the magnetic field [7]. These interactions among the spins aligned in one particular order at a certain temperature below the Curie temperature (T_c_) and above this temperature ferromagnetic domains overcome thermal energy [8]. The very unique characteristic of ferromagnetic property is to have hysteresis loop, featured by the existence of saturation magnetization (M_s_) above which there is no increase of further magnetic property whatsoever be the magnitude of applied magnetic field. Another feature of ferromagnetic materials, remanent magnetization (M_r_), stores even in the absence of applied magnetic field, and this property is related with the memory or storage capacity of materials. Further, these ferromagnetic materials are specified with the coercive field (H_c_) which measures the magnitude of reverse direction of the magnetic field to remove all its magnetization effect. These three properties are of prime importance in finding out the potential phase of ferromagnetic material. There is a competition between exchange magnetostatic and anisotropy energies, and there exist the long- and short-order interaction domains [9].

**Fig. 2:**
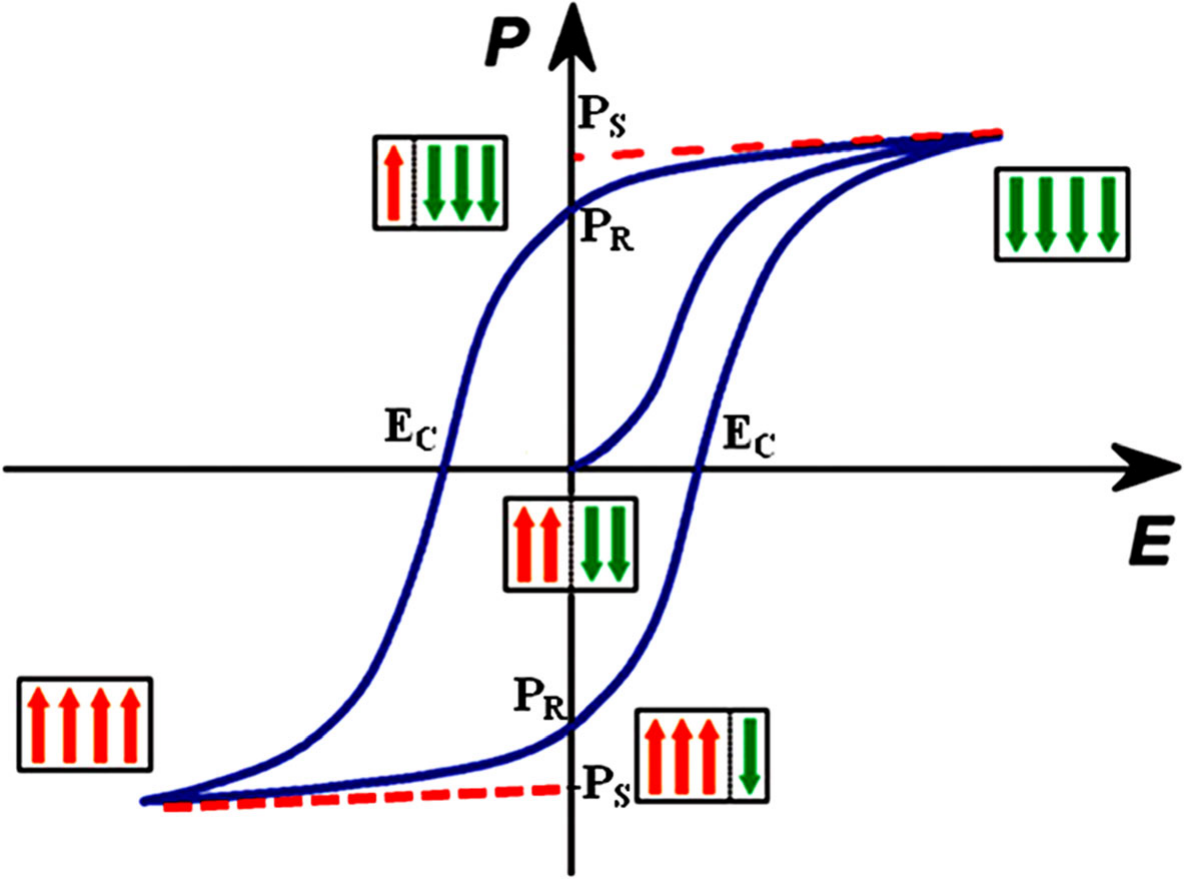
Hysteresis (P-E) curve in ferroelectric materials

Ferroelectric property [10] characterized by the existence of polarization in the presence of applied electric field is analogous with the ferromagnetic property. The difference between the ferroelectric and ferromagnetic lies in the structure of materials but not with atoms, so ferroelectric is an intrinsic property. This property depends on the whole structure and symmetry of compounds and the order, disorder, and displacement of ions that gives rise to the mechanism of ferroelectricity [11–13]. Structured polarization is related with the ferroelectric property that results in the hysteresis loop formed from electric domains. There is a certain temperature below which the phase change from paraelectric to ferroelectric called transition temperature, that in turn depends on the nature of materials. These mini domain characteristics of hysteresis are shown in Fig. 2 and in some manner match with the magnetic hysteresis loop. By plotting a graph between electric polarization versus applied electric field, a loop-like structure was formed with saturation polarization (Ps), remanent polarization (Pr). and coercive field (Hc) [14]. Here, the domain starts to align in positive field direction that gives rise to rapid polarization and reaches to maximum polarization called saturation polarization, and beyond this, there is no further increase in the value of polarization. Further, if the applied field is reversed, polarization tends to decrease and reaches to a particular value where the applied field is zero. Remanent polarization (residual polarization in the material when the electric field is totally removed) is the measure of retaintivity or remanence of the materials used specifically for memory and storage capacity. In order to attain zero polarization, the applied electric field must be further decreased. The magnitude of the applied electric field where the whole polarization becomes zero is called the coercive field. These values are characteristics of hysteresis that depends on the structure, nature, and size of ferroelectric materials [15].

## Multiferroic: a Unique and Novel Property [16]

**Fig. 3:**
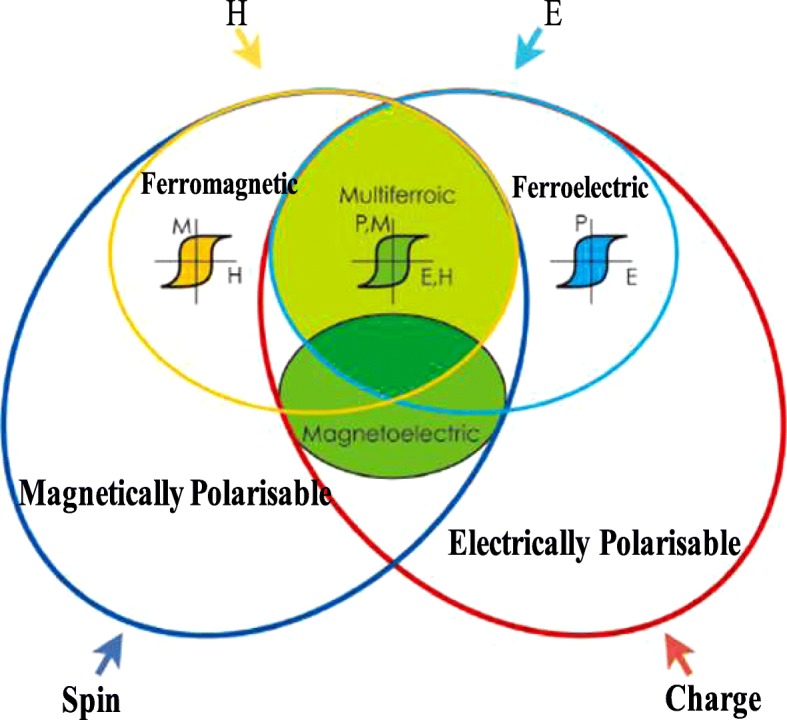
General classification of multiferroic materials. Adapted from Eerenstein et al. [21]

The concept of multiferroic was introduced by H. Schmidt in 1994 [17], and as per the latest definition, multiferroic materials possess simultaneous two or more than two ferroic phases together in a single material [18]. These materials became subject of research to investigate the chemical nature and to study the solid state physics [19]. Bulk research in this field helped to develop a lot of new ideas to utilize in device applications. One of the ideas is to introduce the multiferroic bits that may store information in the form of magnetization and polarization. There are only few materials which have two or more than two ferroic properties and hence the multiferroic materials are rare [20]. This trend of materials having one or more than two properties has been shown in Fig. 3, where it clearly indicates that there are very few materials which show the multiferroic behavior [21]. This is the reason why this field of research is a challenge for the present world and needs to be focussed [22]. Rare existence of multiferroics is related with the mechanism of ferroelectric behavior which demands empty d orbitals, and on the other side, ferromagnetism needs partially filled d orbitals [23, 24]. In order to compensate this sort of controversy and to achieve the multiferroic nature, the structure of the materials needs to be tuned in such a way that an atom may move from the center to form electric dipoles and should be related with magnetic moments. This will lead to either an alternative mechanism for magnetism or ferroelectricity. There are still certain things which may be explored at the nanoscale. The multiferroic nature of nanostructured materials may open new horizons in the applications of making small efficient devices like computer chips, and many more. Recent research is focusing on nano-multiferroic materials for fabrication, design, and applications. The ferroeclectric domain wall structures and the position of magnetic ions plays an important role to get the new functionility for the development of novel devices. The formation, engineering, and application by changing the structures can be used to carry the information in the latest devices. Continuous interest and growing space have been given to multiferroic materials that resulted in the fourth ferroic order called ferrotoroidicity [25, 26] and also determined the electrical conductivity domain walls that are different from bulk materials related with memory properties [27]. Quite a new interesting thing was also observed with the help of film deposition techniques, that the electric field gives the magnetism at room temperature [28]. Although, the multiferroic study has achieved appreciable interest from all the researchers around the world, there is still a poor approach of commercializing the multiferroic materials which need to be accelerated in the near future.

## Various Classes of Multiferroic Compounds on the Basis of Structure

### Bismuth Ferrites (BiFeO_3_ Compounds)

**Fig. 4:**
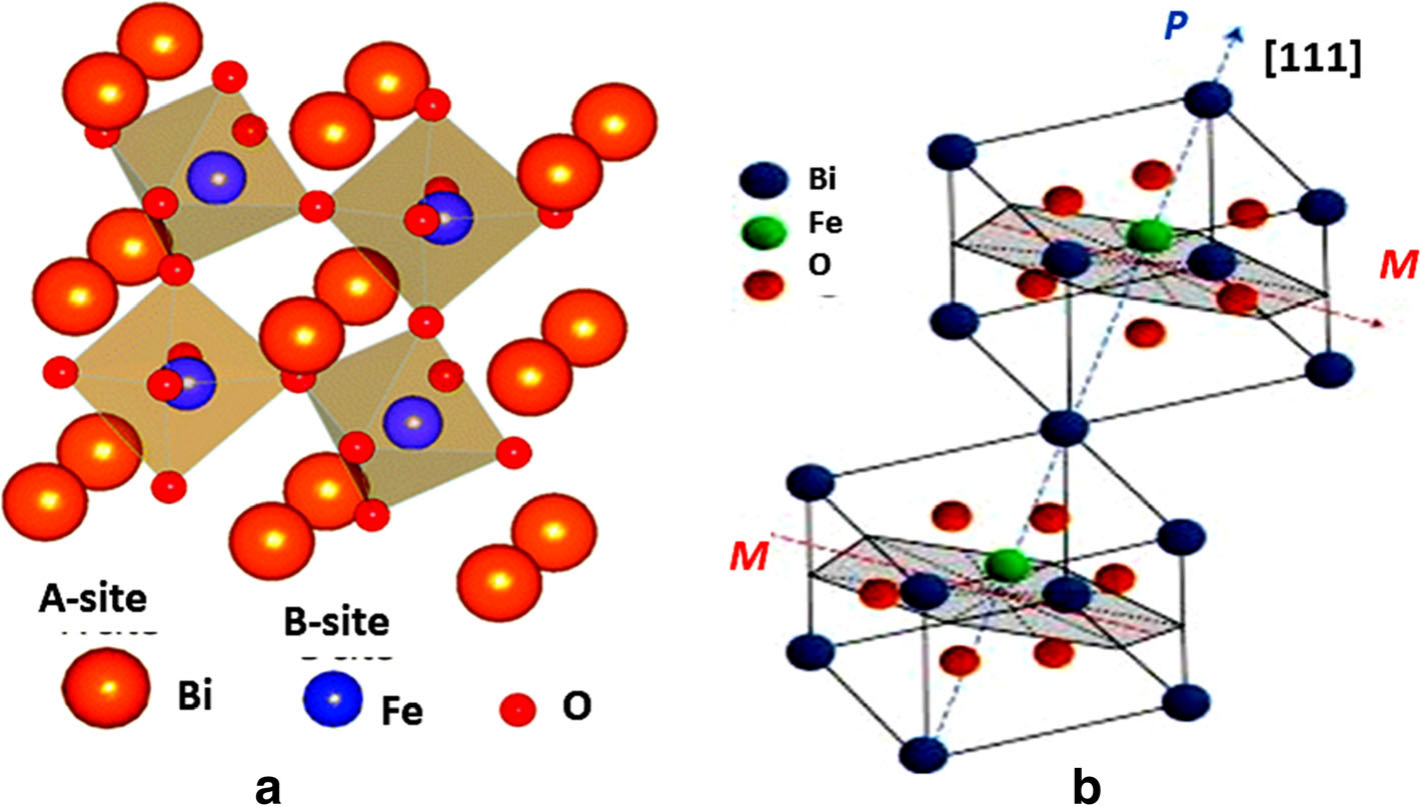
**a** The perovskite crystal structure of BiFeO_3_ adapted from Seidel et al. [28]. **b** Distorted perovskite structure adapted from Ederer and Spaldin [31]

Bismuth ferrite ternary oxides and the derivative compounds are perovskite structures and are promising multiferroic compounds [29]. This ABO_3_ perovskite bismuth ferrite compound has ferroelectricity from the lone pair of electrons at central metal A (Bi^3+^) ion that distorts from the position and the symmetry of the compound lost which provides the ferroelectric property [30]. The cation at the site of B position is Fe^3+^ ion which is small and has unpaired d electrons that give the magnetic properties of BiFeO_3_ compound as shown in Fig. 4 [31]. Here, it can be concluded that polarization is caused by Bi^3+^ lone pair electrons present in 6s^2^ orbitals and magnetic property arises from Fe^3+^ ions. The fabrication of BiFeO_3_ nano-compound may lead to a new direction of research that will help to build interesting multiferroic materials. There were issues of leakage current that reduced the electrical parameters of bismuth ferrites and was later improved by the addition of strontium-zirconium ions into the BiFeO_3_-BaTiO_3_ composites. Further, phase structure, surface texture, and electrical properties were also studied systematically [32]. Much research was carried out in ferroelectric perovskite BiFeO_3_ for many application purposes, but has rarely been investigated for the energy conversion of tiny mechanical motions in electricity in spite of its large theoretical remnant polarization. But there was one report which showed that BiFeO_3_ nanomaterials have such a potential for large-scale lead-free piezoelectric nanogenerator and these nanoparticles were synthesized by a sol-gel process [33]. Bi_5_Ti_3_FeO_15_ (BTF) multiferroic lead-free nanofibers were fabricated by electrospinning and exhibit an effective micro-piezoelectric coefficient with benign micro-ferroelectricity [34]. Further, the coupling behavior between macro-ferroelectric and magnetoelectric was found by non-sintering and pressing for the first time and is smaller than Bi_5_Ti_3_FeO_15_ ceramic. The magnetic moments of BiFeO_3_ were balanced each other by two Fe ions spinning in the opposite direction within the cell, and the band gap was found around 20.5 eV [35]. Density of states were analyzed that indicates that the valence band consists of Fe-d and O-p states, while the conduction band is composed of Fe-d and Bi-p states. The dielectric function, absorption, refractive index, extinction coefficient, reflectivity, and electron energy loss were also reported for BiFeO_3_.

### Yttrium Magnetite (YMnO_3_) Compounds

**Fig. 5:**
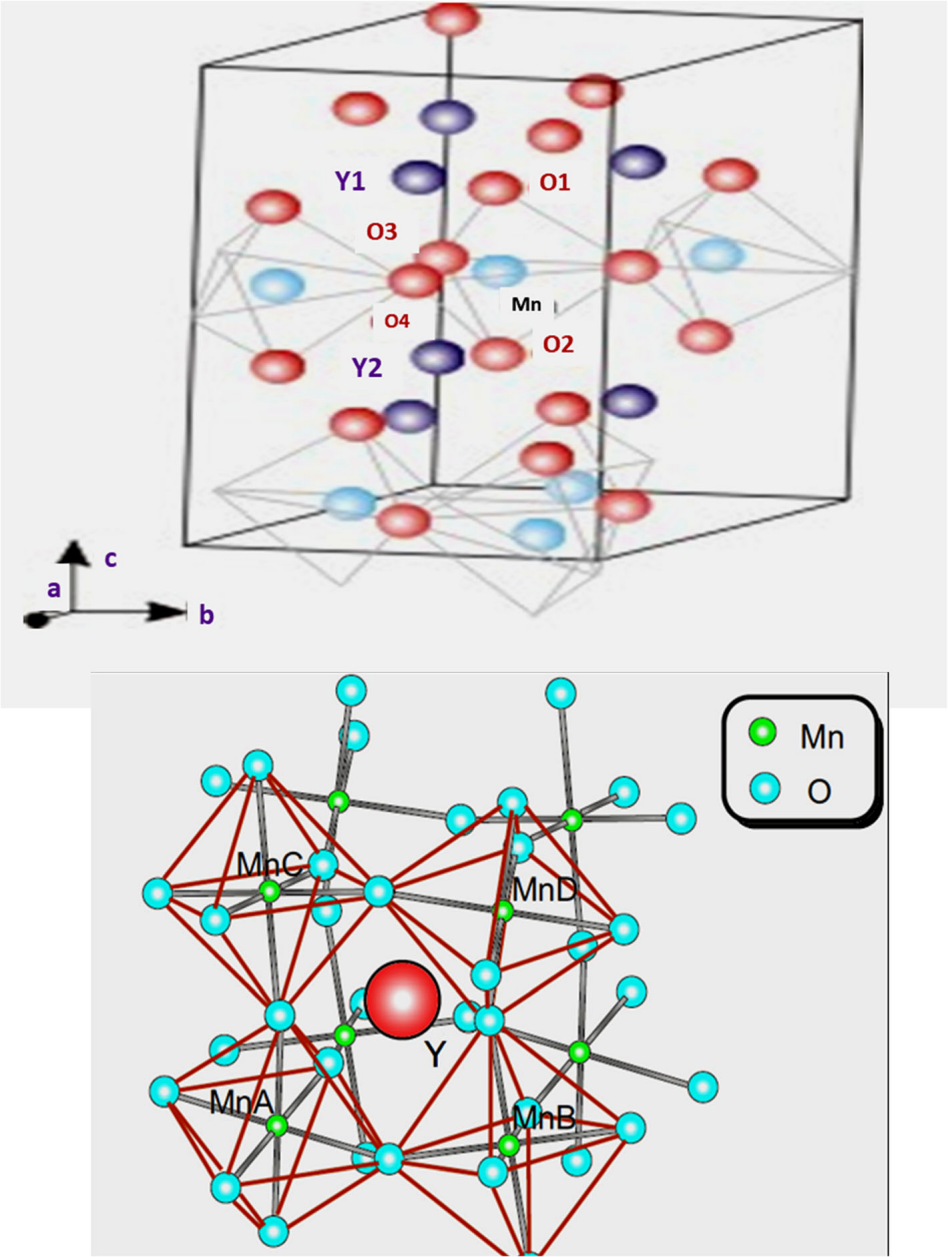
Crystal structure of YMnO_3_ featuring layers of MnO_5_ polyhedra and Y atom in between the layers. Adapted from Wadati et al. [38]

**Fig. 6:**
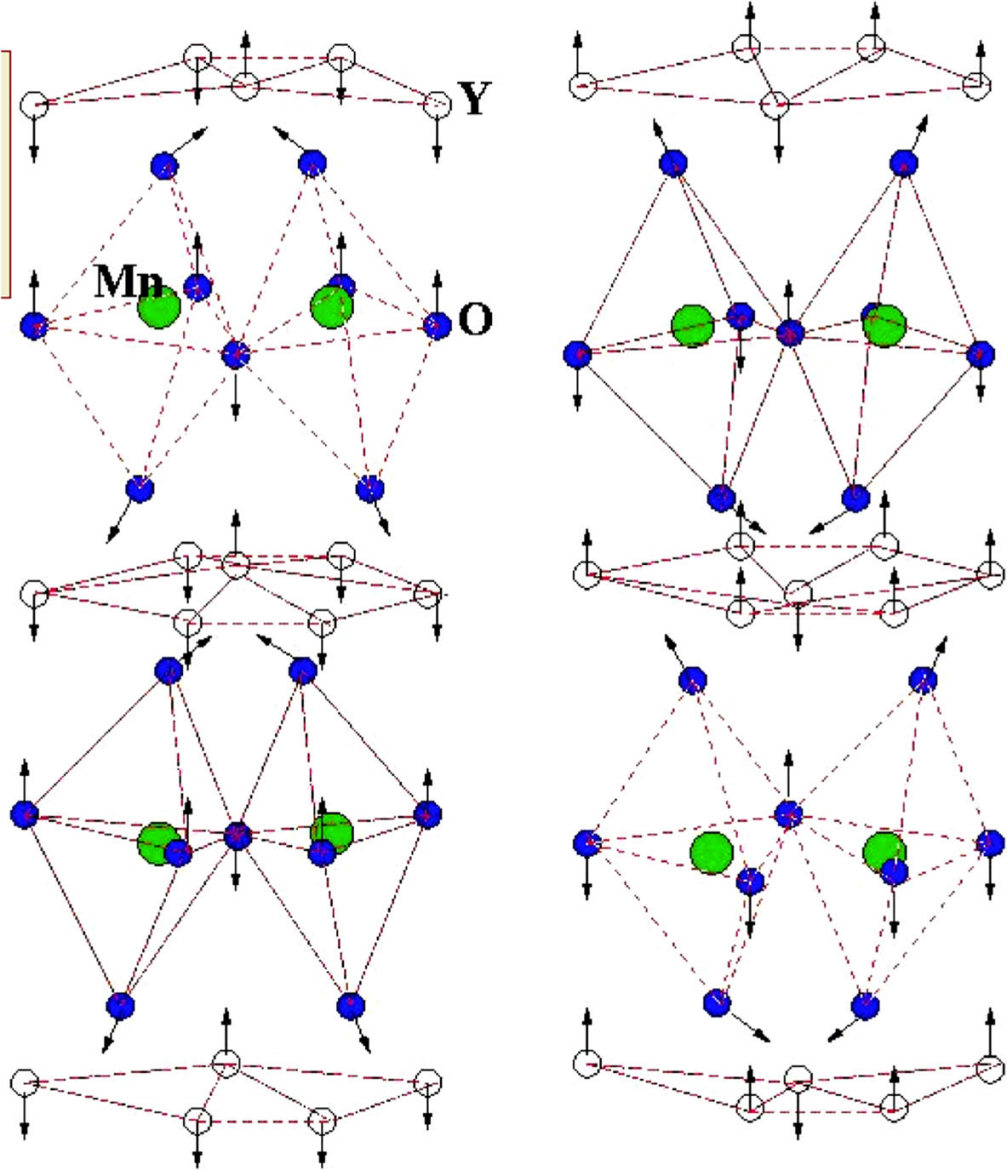
Three-dimensional schematic view of YMnO_3_ in the polarized states. Adapted from Spaldin et al. [39]

It seems that YMnO_3_ compound has the same perovskite ABO_3_ type structure, but it has a different crystal structure and electronic arrangements. In contrast to the conventional perovskites, hexagonal manganites have their Mn^3+^ ions with 5-fold coordination, located at the center of an MnO_5_ trigonal bi-prism. R ions, on the other hand, have 7-fold coordination unlike the cubic coordination in perovskites. The layer of Y^3+^ ions differentiates the two-dimensional MnO_5_ biprism as shown in Fig. 5, which represents the YMnO_3_ unit cell showing ionic structures. A new concept of antiferromagnetic ferroelectricity was found in YMnO_3_, and the geometric structure leads the ferroelectric properties which couples with the magnetic property of YMnO_3_ compound [36]. The tilting of MnO_5_ trigonal biprism results in the loss of inversion symmetry in the structure that leads out the ferroelectric properties of YMnO_3_-type compounds [37]. The coupling between the ferroelectricity and magnetic order is quite unlike, and this is the main reason why magnetoelectric coupling could not be possible in such type of materials. But the ion movements in the tilting-layered MnO_5_ polyhedra lead to the net polarization effect [38, 39] as shown in Fig. 6. It was also reported that hexagonal YMnO_3_ nanofibers prepared by the sol-gel method and the prepared spun fibers were dried at 125 °C with uniform diameter [40]. In an increase in temperature of the prepared sample, there was an adequate change in morphology and diameter range with homogenous chemical constituents over its length.

### Rare Earth (RMO_3_, M = Fe, Cr, Mn ) Multiferroic Compounds

**Fig. 7:**
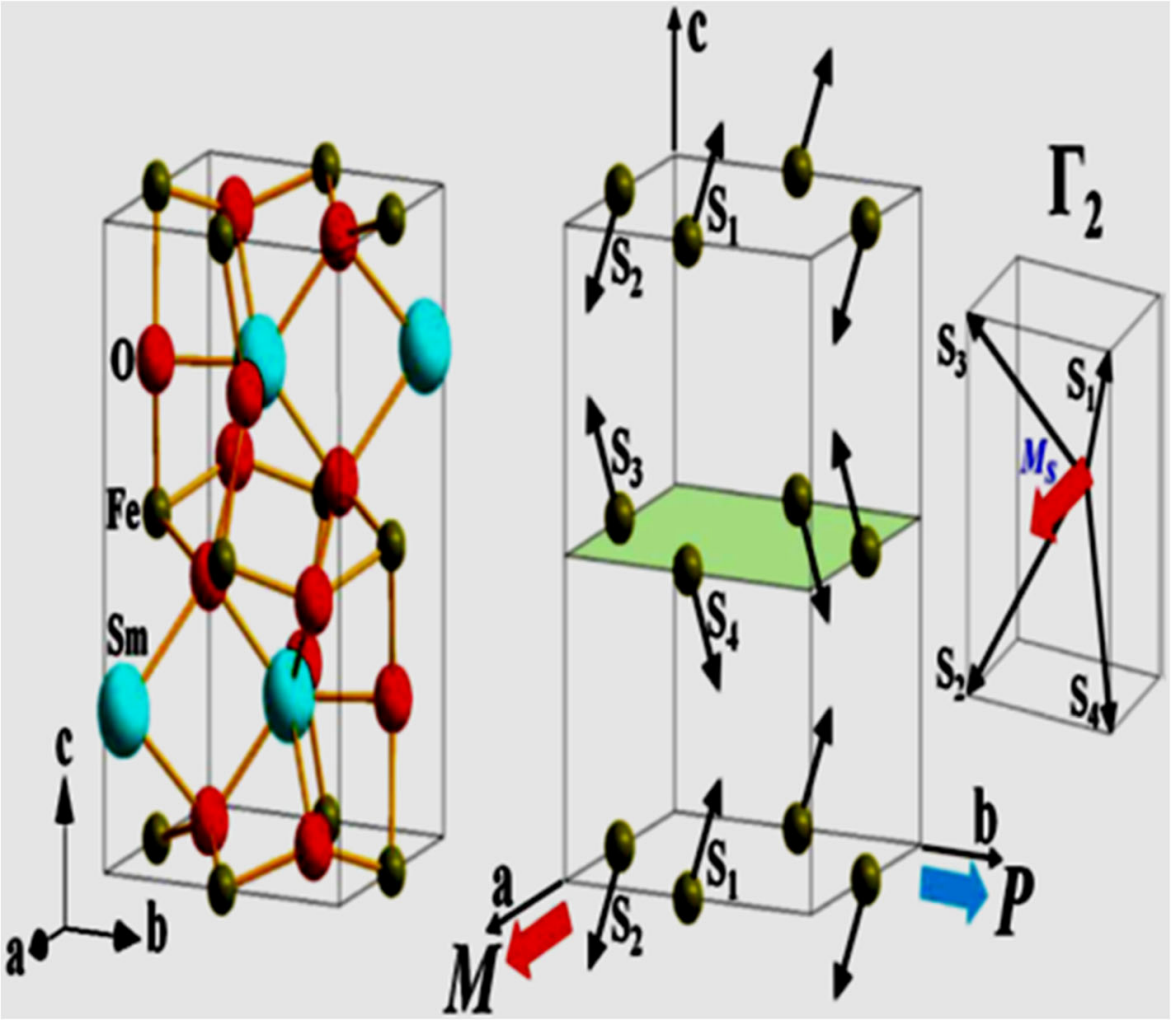
Crystal structure and magnetic spectra of orthorhombic SmFeO_3_. Adapted from Scoot et al. [44]

The latest research found that rare earth metal ternary oxides that may contain iron, manganese, and chromium elements at the B site show multiferroic properties in which weak ferromagnetic is accompanied by the room temperature ferroelectric behavior [41]. In case of RFeO_3_ compounds, the structure of such type of compounds is orthorhombic unit cells [42] with distorted perovskite structure. This distortion is just because of rare earth ion R^3+^ positions and the presence of Fe^3+^ ions in an octahedral environment. Such structures have FeO_6_ octahedra in the three dimension, one of the O^2-^ ions forms one common apex between the two octahedra, and the two iron atoms provide the superexchange bond through O^2-^ ions. In this concept, the Fe atoms are slightly canted that results in the weak ferromagnetic interactions [43]. Since the RFeO_3_ compounds are included in the family of centrosymmetric ferrites, there still exists the room temperature ferroelectric property. This unusual behavior can be explained with the literature which reported a SmFeO_3_ compound where the non-equivalent spins are responsible for the induced ferroelectric property and were given the name of antiferromagnetic ordering-induced ferroelectricity [44] which has been shown in Fig. 7.

**Fig. 8:**
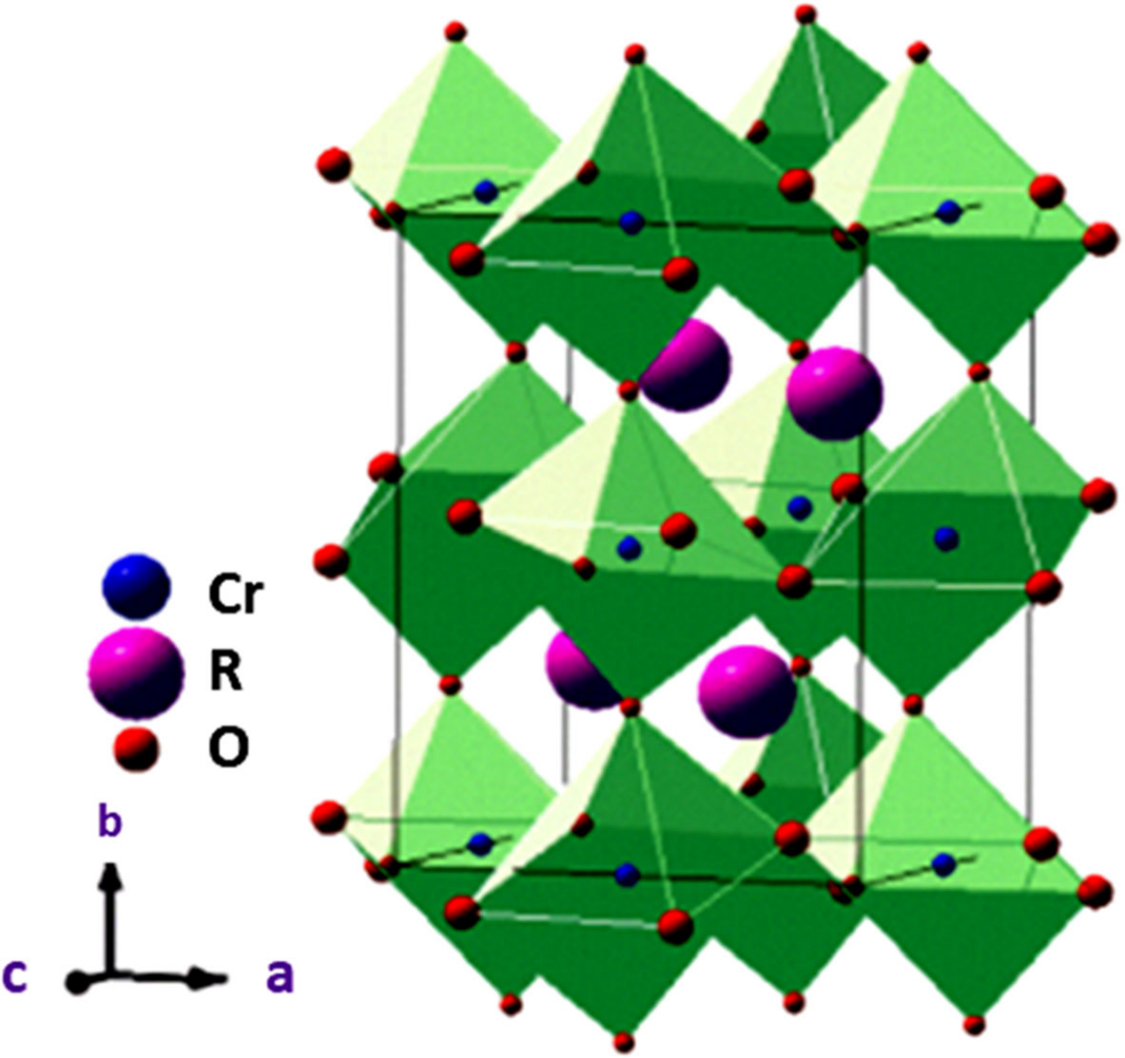
Distorted orthorhombic perovskite crystal structure of RCrO_3_. Adapted from Fender et al. [45]

The second class of rare earth multiferroic oxides is chromium-based RCrO_3_ compounds. In place of FeO_6_ structure, antiphase tilting of CrO_6_ octahedra as shown in Fig. 8 was present in orthorhombic (RCrO_3_, R = Y, Gd, Tb) multiferroic compounds. The polarization of ferroic nature couples with the magnetic ordering of Cr ion sublattices, and the well-known interaction Dzyaloshinskii-Moriya (DM) gives rise to the weak ferromagnetic properties of Cr^3+^ ions [45]. GdCrO_3_ compounds, the magnetic moment of Cr ions, are antiparallel to its nearest cations and are represented by G-type configuration. The class of ferroelectricity of RCrO_3_ compounds is still not explained properly, while it was assumed that off-centring distortion has been proposed for the origin of ferroelectric behavior. This kind of mechanism was reported in bulk, nano, thin films of RCrO_3_ compounds [46–48]. In the presence of applied magnetic field, the strength of polarization can be varied in case of GdCrO_3_ compounds. YCrO_3_ is orthorhombic but still is ferroelectric as the Cr atoms are displaced from the position in a particular direction which results in the polarization. This shows the new concept that can be visualized by many unusual properties of multi-functionalized materials.

Cubic GdFeO_3_ particle by a simple hydrothermal synthesis route and its photoluminescence and magnetic properties were investigated [49]. Through the investigation of the photoluminescence and magnetic properties, the orthorhombic cubic GdFeO_3_ particles exhibited very good doped luminescence, which emits different colored light when doped with different rare earth elements. The GdFeO_3_ particles contain paramagnetic properties. It could be an excellent luminescence and magnetic material. High magnetoelectric coupling by using one single crystal of DyFeO_3_ and GdFeO_3_ has been reported before, but the multiferroic nature occurs only at very low temperature [50]. Solid-state powder synthesis of GdFeO_3_ and GdCrO_3_ involves the extensive mechanical grinding of the required oxides (Gd_2_O_3_, Fe_2_O_3,_ and Cr_2_O_3_) at high enough calcination temperature ∼ 1800 °C. A simple sonochemical method for the synthesis of nanoparticles of a series of rare earth orthoferrites was reported. This sonochemical process is enabling the synthesis of nanoparticles of the rare earth orthoferrites at a substantially lower calcination temperature by using simple precursors, iron pentacarbonyl, and rare earth carbonates. It is particularly noteworthy that the cogeneration of the garnet phase has not been observed, as is usual with the conventional methods. The drastic reduction in the calcination temperature could be due to the ultrasonic generation of amorphous iron oxide from Fe(CO)_5_. Nanosized GdFeO_3_, ErFeO_3_, TbFeO_3_, and EuFeO_3_ were prepared by this method, and their magnetic properties were also studied in detail [51]. Highly crystalline orthoferrite nanoparticles (type La_1−x_ Gd_x_FeO_3_, where x = 0 to 1) were prepared using the self-combustion method. Our particular interest is in the characterization of the structural and magnetic properties of given compounds with a strong emphasis on the role of Gd^3+^ ions in the modulation of the structure and magnetic response [52]. Perovskites with composition MFeO_3_ are a class of materials having potential applications such as catalysts [53], sensors, [54] semiconductors, and [55] magnetic and magneto-optical materials [56]. The phase-selective synthesis of LnFeO_3_ (Ln = rare earth) is challenging because there are undesired coexisting phases [57, 58]. Visible-light-driven Gd_2_Ti_2_O_7_/GdCrO_3_ composite for hydrogen evolution has been reported, and a series of Gd_2_Ti_2_O_7_/GdCrO_3_ composites are prepared by solid-state combustion. The photocatalytic activity of the composites is examined towards hydrogen production without using any co-catalyst under visible light illumination. The rate of formation of hydrogen is measured by the photocatalytic activity measurement device and gas chromatography (GC). The highest efficiency is observed over the composite GTC (Cr:Gd:Ti = 1:1:1). On the basis of photocurrent measurements and PL, a mechanism for the enhanced photocatalytic activity has been discussed [59]. Unusual magnetic properties of nanocrystalline orthoferrite, GdFeO_3_, synthesized by conventional solid-state reaction (SSR) route based on the stoichiometric mixing of Fe_2_O_3_ and Gd_2_O_3_ have been found in the report [60]. The polycrystalline samples of GdFe_1-x_Ni_x_O_3_(*x* = 0.0, 0.1) are prepared by solid-state reaction route. It was also noticed that Ni^3+^ ion substitution results in lattice contraction and enhancement in a dielectric constant, tangent loss, and AC conductivity [61].

The only available magnetic studies were focused on the Mossbauer spectrometry to probe field-induced SR transitions in DFO [62, 63]. Among these compounds, DFO is the only rare earth orthoferrites that show the Morin transition at 35 K followed by three anomalous transitions at temperatures 77 K, 130 K, and 270 K originating probably due to the field-induced spin reorientation (SR) effect originating from the competing magnetic interaction between Dy^3+^ and Fe^3+^ ions. Microwave-assisted synthesis of rare earth chromites and physical properties were reported. Magnetization measurements showed that the Neel temperature for antiferromagnetic Cr^3+^-Cr^3+^ ordering strongly depends on the RE^3+^ ionic radius and a rich variety of different magnetic spin interactions exists. On sintered pellets the electronic differences at grain boundary and interior bulk material, which gives the two dielectric relaxations monitered by dielectric spectroscopy. X-ray diffraction, Raman spectroscopy, and temperature-dependent dielectric permittivity data do not indicate potential non-centrosymmetry in the crystal or concomitant ferroelectricity. Systematic efforts have been carried out to prepare full series of (RE)CrO_3_ compounds, that may resemble in structure of YCrO_3_ compound. Detailed investigation of the magnetic and dielectric properties and their correlations with a particular focus on possible magnetoelectric or multiferroic behavior as observed was reported [64]. The charge transport properties in (RE)CrO_3_ materials have been claimed to involve p-type semiconductivity with sensitivity towards humidity, methanol, ethanol, and several gases, which is useful for potential sensor applications. [65, 66]. Furthermore, LaCrO_3_ and its doped variants are candidates for application as interconnected materials in solid oxide fuel cells [67, 68] and as catalysts for hydrocarbon oxidation [69]. Rare earth orthoferrites of the type LnFeO_3_ (Ln ¼ Gd, Dy, Sm) are crystallizing the orthorhombically distorted perovskite structure. The presence of electric polarization in the weakly ferromagnetic state of DyFeO_3_ was reported in a polycrystalline sample, [70] wherein ferroelectricity disappears below the spin reorientation temperature. The importance of the local field induced on Dy ions by the weak ferromagnetic moment of the Fe sublattice in the G_4_ structure is revealed by the zero-field [71] Fe Mossbauer spectra of DyCrO_3_. Magnetic susceptibility of heavy rare earth orthochromites at higher temperature [72] and magnetocaloric properties of rare earth-substituted DyCrO_3_ have also been reported [73]. The detailed investigation of magnetic interaction was found in DyCrO_3_ bulk powders [74] using hydrothermal synthesis method. Detailed studies on nanocrystalline CeCrO_3_ were found to exhibit multifunctionalities such as antiferromagnetism, relaxor behavior, and an optical band gap in the visible region. This newly developed synthesis route opens the immense possibilities of preparation of the hitherto unknown Ce^3+^-based mixed oxides, analogous to other rare earth (RE^3+^) counterparts [75]. The field-induced metastable state with electric polar order appears at the magnetic ordering temperatures of Cr^3+^ ions in the weakly ferromagnetic rare earth orthochromites (RCrO_3_, where R is a magnetic rare earth ion), exhibiting a relatively large electric polarization ~ 0.2–0.8 μC/cm^2^, starting at rather high temperatures (~ 120–250 K) corresponding to the Neel temperatures of the Cr subsystem [76]. Static and dynamic magnetic properties and effect of surface chemistry on the morphology and crystallinity of DyCrO_3_ nanoplatelets have been reported [77].

It was also reported that nanosized orthoferrites can be used as photocatalysts in the decomposition of water or the degradation of dyes under light irradiation. This area of research has been enlarged significantly due to the advent of a novel class of oxides exhibiting interesting multiferroic and magnetoelectric properties arising from magnetically induced ferroelectricity. Interestingly, these materials are simple transition metal oxides, most of them possessing the perovskite structure. Novel features of multiferroic and magnetoelectric ferrites and chromites exhibiting magnetically driven ferroelectricity. It has been seen that almost all oxide semiconductor photocatalysts are stable but active under UV light irradiation. Developing a general mild method to prepare rare-earth chromites of uniform crystal size and shape is important for further single crystal related applications. The micrometer-sized single crystals preserve more of the bulk properties compared with their corresponding polycrystalline counterparts acquired with high-temperature treated precursors. Understanding crystal structures and band structures of complex metal oxides is without doubt a key aspect to explore new or improved functionalities. For low-temperature reactions, in particular, the topochemical ones, equally important is the understanding of the factors to direct final structures during a reaction, such as intermediate phase and ion-migration route, by utilizing both kinetic and thermodynamic considerations. In addition, such knowledge, as demonstrated here by the thin film work, will definitely help in developing new ion conductors toward low-temperature applications. The macroporous walls are composed of rare earth orthoferrite nanoparticles, and these hierarchically porous materials show high catalytic activities for the CO+NO reaction, and NO can be fully converted to N_2_ at temperatures as low as 350°C, indicating their potential in the catalytic conversion of automotive exhaust gas and other catalysis-related fields. This synthesis strategy is a facile method for the preparation of hierarchical porous materials and may give us a guideline for the synthesis of functional materials with further catalytic applications [78]. With the development of the automobile industry, automobile exhaust gas has become one of the major sources of air pollution. The control of automobile exhaust pollution is particularly significant for reducing air pollution. TbFeO_3_ compounds which possess space group Pbnm may have antiferromagnetic interactions by the presence of Fe spin ions in one direction and the ferromagnetic in other direction with the (TN) Neel temperature of 650 K [79, 80]. The work that has been found for synthesis characterization and the properties of TbFeO_3_ compound needs to be explored much more as compared to other rare earth oxide ferrites [81–83]. The choice to select the atom at A site has become an important concern and may be related with leakage and the loss of multiferroic nature. The structures and magnetic phase transitions in the Mn-doped orthoferrite TbFeO_3_ studied by neutron powder diffraction have been reported [84].

## Ternary Metal Oxide Nano-Material Applications

The application of multiferroic materials is expected from the data values of polarization and magnetization with the existence of magnetoelectric coupling. This could be the main reason that these interesting materials have to be considered in today’s research of solid state physics and chemistry and may utilize in electronic memory and optical transducer devices [85–87]. These materials not only possess the memory capacity but may also have sensing properties with magnetic and electronic nature. Multiferroic materials need to be explored further for novel devices by reducing thermal noise for the use of capacitive reading and can replace the magnetoresistive materials [88]. These magnetic-related properties are more sensitive than conventional resistive measurements that allow the magnetic bit density and posses four state memory property [89] which was demonstrated by the encoded information with the help of polarization and magnetization that too measured by resistance measurements. Many nanostructured and nanoscale coating materials have been suggested as possible friction modifying agents, such as carbides, nitrides, metals, and various ceramics. In conclusion, nanotechnology helps to create vehicles possessing properties to endure the harsh conditions of space. Both magnetic and electric properties have the advantage to store data that could be written electrically and read magnetically. This advantages of multiferroic avoid the generation of large load fields to write and read problems [90]. Fe-RAMS devices have been designated using the concept of ferroelectric writing and ferromagnetic reading, and the retained non-volatile memory has been increased thousand times and even more by the use of the same materials at nano-regime. Thus, nanomaterials having such multiferroic properties have tremendous applications in all devices such as memory, sensory, and optical. The size-dependent unconventional multiferroic compounds in nanodots having emerging magnetic properties along with ferroelectric properties were reported. The nanometric size with nonstoichiometric induces the ferromagnetism with host ferroelectric phase and is susceptible to surface morphology that enables to control the properties at the nanoscale [91]. The magnetoelectric coefficients increase on reducing the particle size and could be related with high strain and suppression of spin spiral structure. The electric and magnetic properties of Bi_0.90_Tb_0.10_FeO_3_ nanoparticles depend on the particle sizes and were revealed high as the particle size decreases [92]. In case of Bi_2_Fe_4_O_9_ polycrystalline, the magnetic and ferroelectric properties were investigated with different grain size [93]. Grain size effects the decrease of the ferromagnetic part, but the antiferromagnetic component part dominates as the size increases and shifts the Neel temperature to a higher value. Ferroelectric properties lead to non-volatile data storage devices and high demand in ultrafast electronic instruments which are portable and have high density to storage with less power consumption. Therefore, it is essential to fabricate and to develop such multiferroic nanomaterials which have high sensitivity and efficiency and have a bulk of applications in all segments of machines.

## Conclusion

Multiferroic ABO_3_ type compounds have been focused in the present review based on their structure, composition, and contribution to ferroelectric and ferromagnetic properties. The various factors that improve or decrease the multiferroic properties were taken into consideration. The significant efforts for the synthesis and development of ABO_3_-based perovskite multiferroic compounds were also mentioned. We attempted to give the outline of specific ternary metal oxide multiferroic compounds that may include bismuth ferrites, yttrium magnates, and rare earth oxides. These ABO_3_ multiferroic compounds have a lot of applications such as in microelectronic devices, sensors, and storage devices. It is not impossible but rather it is hard to get the breakthroughs of multiferroic compounds in the field of commercialization, and this kind of expectation is expected with the help of research that these productive insights will come soon. It could take further time to develop new materials to achieve the applications in other areas such as magnetoelectric sensors and magnetometers or antennas. There is always a room for improvement of these multiferroic materials and has a lot of market potential in magnetic anomaly detection, navigation, and biomagnetic sensing. If these multiferroic materials are successfully prepared, developed and then commercialized, it will be a breakthrough or huge impact on everyday life and people may choose to stay in academia, join industry, or even start up new businesses.

## Abbreviations

AC: Alternating current
DFO: Dysprosium ferrite oxides
DM: Dzyaloshinskii-Moriya
GC: Gas chromatography
Hc: Coercive field
M_r_: Remanent magnetization
MRI: Magnetic resonance imaging
M_s_: Saturation magnetization
Pr: Remanent polarization
Ps: Saturation polarization
RE: Rare earth
SR: Spin reorientation
SSR: Solid state reaction
TC: Curie temperature
TN: Neel temperature
